# Psychomotor slowing alters gait velocity, cadence, and stride length and indicates negative symptom severity in psychosis

**DOI:** 10.1038/s41537-022-00324-x

**Published:** 2022-12-30

**Authors:** Melanie G. Nuoffer, Stephanie Lefebvre, Niluja Nadesalingam, Danai Alexaki, Daniel Baumann Gama, Florian Wüthrich, Alexandra Kyrou, Hassen Kerkeni, Roger Kalla, Sebastian Walther

**Affiliations:** 1 Translational Research Center, University Hospital of Psychiatry and Psychotherapy, University of Bern, Bern, Switzerland; 2 Graduate School for Health Sciences, University of Bern, Bern, Switzerland; 3 Klinik Sonnenhalde AG Psychiatrie und Psychotherapie, Riehen, Switzerland; 4 Department of Neurology, Inselspital, Bern University Hospital, University of Bern, Bern, Switzerland

**Keywords:** Schizophrenia, Psychosis

## Abstract

Schizophrenia is a severe mental disorder, in which 50% of the patients present with motor abnormalities such as psychomotor slowing. Slow spontaneous gait has been reported in schizophrenia. However, comprehensive objective instrumental assessments of multiple gait conditions are missing. Finally, the specific gait patterns of subjects with psychomotor slowing are still unknown. Therefore, this study aimed to objectively assess multiple gait parameters at different walking conditions in patients with schizophrenia with and without psychomotor slowing. Also, we hypothesised gait impairments to correlate with expert ratings of hypokinetic movement disorders and negative symptoms. We collected gait data (GAITRite®) in 70 patients with psychomotor slowing (SRRS (Salpetriere retardation rating scale) ≥15), 22 non-psychomotor slowed patients (SRRS < 15), and 42 healthy controls. Participants performed four walking conditions (self-selected speed, maximum speed, head reclined, and eyes closed) and six gait parameters were extracted (velocity, cadence, stride length, functional ambulation profile (FAP), and variance of stride length and time). Patients with psychomotor slowing presented slower velocity, lower cadence, and shorter stride length in all walking conditions compared to healthy controls, with the non-slowed patients in an intermediate position (all F > 16.18, all *p* < 0.001). Secondly, slower velocity was associated with more severe hypokinetic movement disorders and negative symptoms. In conclusion, gait impairments exist in a spectrum with healthy controls on one end and patients with psychomotor slowing on the other end. Patients with psychomotor slowing are specifically impaired when an adaptation of gait patterns is required, contributing to the deleterious effects of sedentary behaviours.

## Introduction

Schizophrenia is a severe and chronic mental illness, characterised by positive (e.g. delusions, hallucinations) and negative symptoms (e.g. avolition, social withdrawal), poor cognition, mood alterations, and motor abnormalities (e.g. psychomotor slowing, stereotypy, catatonia)^1–3^. In the past, motor abnormalities were mainly attributed to side effects of antipsychotic medication^1,4,5^. However, spontaneous motor abnormalities appear at all stages of the illness and may interact differentially with antipsychotic medication^5–7^. In fact, 67% of antipsychotic-naïve patients^8^ and 80% of chronic schizophrenia patients^9^ exhibit at least one motor symptom. Motor abnormalities are linked to increased sedentary behaviour^10–12^, reduced quality of life^13^, and predict a more severe course of illness or poorer treatment outcome^14–17^.

Psychomotor slowing is one of the most frequent motor abnormalities in schizophrenia spectrum disorders^1,10,11^, impairing both fine and gross motor behaviours, such as slowed writing, slowed gait^18,19^, aberrant posture^19^, increased sway^19^, and diminished speech, facial, and gestural expression^10,20^. Psychomotor slowing, parkinsonism, and catatonia are hypokinetic movement disorders, all of which are associated with low physical activity and increased sedentary behaviour in schizophrenia^11,21–24^.

Motor abnormalities pose a massive burden on subjects with schizophrenia^24^, and are poorly addressed by antipsychotics^6^, thus calling for novel specific treatments. To develop such treatment strategies, objective measurement of psychomotor abnormalities is critical. Modern technologies such as accelerometers^11^, three-dimensional ultrasonic movement analysis^25^, motion capture technology^19^, and pressure sensitive walkways (e.g. GAITRite®)^26^ offer new opportunities to capture gross motor behaviour and especially gait in various situations. These methods are objective, reliable, and yield more extensive information on gait than a visual assessment by clinicians^27^.

While prior research has established reduced velocity (distance covered in centimetres per second) in patients with schizophrenia, data on other gait parameters remains equivocal^18,19,28–32^. For example, slower velocity has been reported to be attributed to lower cadence (steps per minute)^32^, shorter stride length^18,19^, or both^31^. Furthermore, prior reports failed to consider and evaluate the effects of motor abnormalities in their sample of patients with schizophrenia using comprehensive and objective methods of gait analysis. Finally, it remains unknown whether individuals with schizophrenia have consistent gait abnormalities when walking in different situations, such as walking fast or walking while being distracted.

Thus, the current report aimed to provide a comprehensive evaluation of gait in schizophrenia using six different parameters (velocity, cadence, stride length, functional ambulation profile, variance in stride time, and variance in stride length), and four different conditions (two ecological conditions [self-selected (Self_Speed) and maximum walking speed (Max_Speed)] and two distractor conditions [walking with head reclined (Head_Rec) and walking with eyes closed (Eyes_Closed)] in patients with schizophrenia and psychomotor slowing (PS, *N* = 70, Salpêtrière Retardation Rating Scale^33^ (SRRS) ≥ 15), patients with schizophrenia and without psychomotor slowing (NPS, *N* = 22, SRRS < 15), and healthy controls (HC, *N* = 42); for details, see Methods section below.

We hypothesised that PS present specifically altered gait patterns within the four different conditions compared to NPS and HC. In addition, we hypothesised that within patients with schizophrenia, gait parameters would correlate with the severity of psychomotor slowing and other hypokinetic motor abnormalities.

## Results

### Demographics and clinical data

The three groups did not differ in age, sex, or BMI (Table 1). Likewise, PS and NPS showed no difference in dosage of current medication, mean Positive and Negative Syndrome Scale^34^ (PANSS) positive score, and Brief Negative Symptom Scale^35^ (BNSS) avolition subscore. Patients with psychomotor slowing had higher PANSS negative, general, and total scores than the patients without. They also had higher ratings on parkinsonism (Unified Parkinson Disease Rating Scale^36^, UPDRS), catatonia (Bush-Francis Catatonia Rating Scale^37^, BFCRS), and negative symptoms measured by the BNSS, especially in the subdomain of emotional expressivity. Males and females did not differ in clinical parameters (Supplementary Table S1).

**Table 1:** Demographic and clinical characteristics.

	HC	NPS	PS	Comparison
N	42	22	70	—
Age (years)	36.71 (12.93)	32.73 (10.48)	35.40 (11.94)	*F*(2,131) = 0.8, *p* = 0.46
BMI	23.78 (4.02)	25.30 (4.89)	25.41 (4.91)	*F*(2,131) = 1.7, *p* = 0.18
Education (years)	16.33 (3.35)	12.91 (1.95)	13.01 (2.36)	*F*(2,131) = 22.9, *p* < 0.001* Contrasts: HC vs. PS: *F* = 3.3, *p* < 0.001* HC vs. NPS: *F* = 3.4, *p* < 0.001* NPS vs. PS: *F* = −0.1, *p* = 0.99
Sex (N / %) female	21 (50%)	12 (55%)	35 (50%)	*χ2* (2) = 0.6, *p* = 0.74
Medication (OLZ eq.)	—	14.32 (10.38)	17.22 (10.73)	*W* = 642, *p* = 0.24
Duration of illness (years)	—	6.55 (7.39)	9.8 (9.43)	*W* = 587, *p* = 0.093
Number of episodes	—	3.33 (2.58)	5.0 (4.69)	*W* = 579, *p* = 0.14
PANSS total	—	65.0 (14.67)	79.90 (16.32)	*W* = 401, *p* < 0.001*
PANSS neg.	—	15.14 (4.05)	23.71 (6.16)	*W* = 183, *p* < 0.001*
PANSS pos.	—	16.23 (4.66)	16.01 (5.08)	*W* = 817, *p* = 0.67
PANSS general	—	33.64 (8.58)	40.17 (9.07)	*W* = 464, *p* = 0.005*
SRRS	—	8.36 (2.82)	23.40 (5.60)	*W* = 0, *p* < 0.001*
mSRRS	—	2.86 (1.70)	10.43 (3.0)	*W* = 24.5, *p* < 0.001*
BFCRS	—	1.27 (1.72)	5.31 (4.0)	*W* = 223, *p* < 0.001*
UPDRS	—	8.50 (5.85)	20.24 (11.1)	*W* = 271, *p* < 0.001*
BNSS	—	26.14 (10.38)	41.7 (14.17)	*W* = 279, *p* < 0.001*
BNSS expressivity	—	1.73 (1.03)	3.48 (1.36)	*W* = 236, *p* < 0.001*
BNSS avolition	—	2.64 (1.31)	3.24 (1.53)	*W* = 575, *p* = 0.072

For each continuous measure the mean is indicated with the standard deviation in brackets. For comparison between three groups an ANOVA is calculated (F). For comparison between two groups a Mann–Whitney U-test is performed (W). For sex a Chi-square test is calculated (χ2).

*HC* healthy controls, *NPS* non-psychomotor slow, *PS* psychomotor slow, *N* number of participants, *BMI* Body Mass Index, *f* female, *OLZ eq*. Olanzapine-equivalent (mg/day), *PANSS* Positive and Negative Syndrome Scale, *neg.* negative, *pos.* positive, *SRRS* Salpêtrière Retardation Rating Scale, *mSRRS* motoric part of the Salpêtrière Retardation Rating Scale, *BFCRS* Bush-Francis Catatonia Rating Scale, *UPDRS* Unified Parkinson Disease Rating Scale Part III, *BNSS* Brief Negative Symptom Scale.

*p ≤ 0.05.

### Gait parameters

**Fig. 1:**
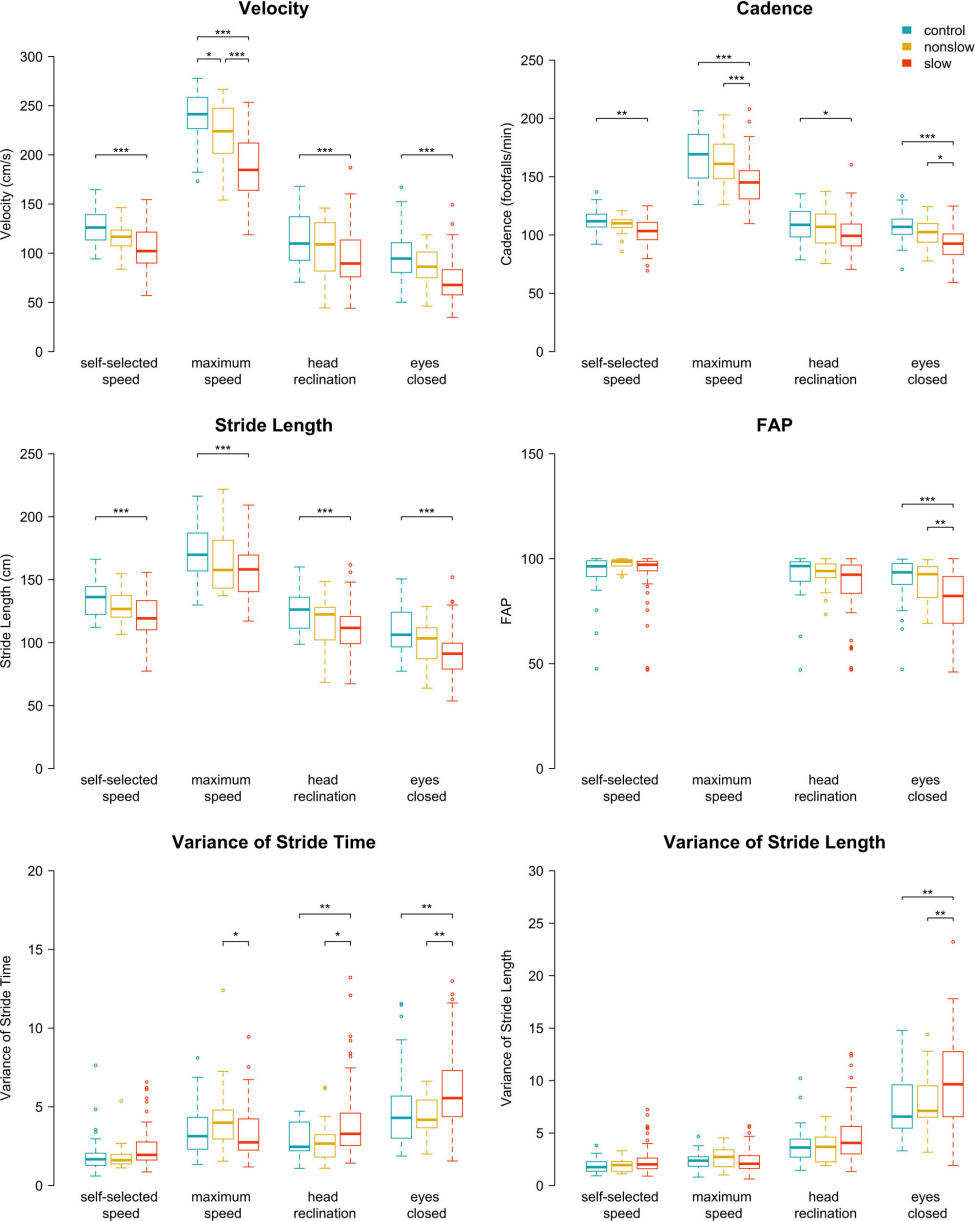
Boxplots per group and condition for each gait parameter. Box represents the interquartile range (IQR; distance between first quartile Q1 to third quartile Q3) with median in between. Whiskers represent the outermost values within Q1 − 1.5×IQR and Q3 + 1.5×IQR. Outliers are plotted individually as circles. Significant differences between groups within the condition are marked with brackets. For comparisons with healthy controls, the results of the main ANCOVA are depicted and for comparisons between patient groups, the results of the second ANCOVA are illustrated. FAP functional ambulation performance score; blue (left): healthy controls; yellow (middle): non-psychomotor slow; red (right): psychomotor slow. *p ≤ .05; **p ≤ .01; ***p ≤ .001.

The ANCOVAs across three groups with age and BMI as controlling variables demonstrated significant group × condition interactions, for all the gait parameters except for stride length (only significant main effects) (Fig. 1, Table 2, Supplementary Fig. S1). Compared with PS, the HC presented with significantly higher velocity, cadence, and stride length in every condition. Also, the HC showed higher functional ambulation performance (FAP) and smaller variance of stride length than the PS only during the Eyes_Closed condition, and smaller variance in stride time for both distractor conditions. For all the conditions and gait parameters, the NPS had intermediate performance between HC and PS, with significant differences compared to the PS group only in some of the Max_Speed or Eyes_Closed conditions.

**Table 2:** Effect of condition and group on gait performance for each gait parameter.

	Main ANCOVA (controlling for age, BMI)	Condition	Mean (sd)	Contrasts	ANCOVA patients (controlling for age, BMI, medication)	Contrasts PS vs. NPS
Velocity (cm/s)	Interaction: Condition x Group *F*(6,393) = 8.0, *p* < 0.001, *p*_*corr*_ < 0.001* Main effects: Group: *F*(2,129) = 36.2, *p* < 0.001* Condition*: F*(3,393) = 1145, *p* < 0.001* Covariates: Age: *F*(1,129) = 2.2, *p* = 0.14 BMI: *F*(1,129) = 2.2, *p* = 0.14	Self_Speed	HC: 127 (17.6) NPS: 116 (16.0) PS: 104 (21.5)	PS vs. HC: *p* < 0.001* NPS vs. HC: *p* = 0.27 PS vs. NPS: *p* = 0.14	Interaction: Condition x Group *F*(3,270) = 5.9, *p* < 0.001, *p*_*corr*_ = 0.001* Main effects: Groups: *F*(1,87) = 14.0, *p* < 0.001* Condition: F(3,270) = 717, *p* < 0.001* Covariates: Age: *F*(1,87) = 3.8, *p* = 0.055 BMI: *F*(1,87) = 1.2, *p* = 0.28 Medication: *F*(1,87) = 1.4, *p* = 0.24	*p* = 0.09
		Max_Speed	HC: 237 (26.7) NPS: 221 (31.2) PS: 187 (30.2)	PS vs. HC: *p* < 0.001* NPS vs. HC: *p* = 0.037* PS vs. NPS: *p* < 0.001*		*p* < 0.001*
		Head_Rec	HC: 114 (25.5) NPS: 104 (28.7) PS: 94 (26.9)	PS vs. HC: *p* < 0.001* NPS vs. HC: *p* = 0.33 PS vs. NPS: *p* = 0.25		*p* = 0.17
		Eyes_Closed	HC: 98 (23.8) NPS: 84 (18.7) PS: 72 (21.6)	PS vs. HC: *p* < 0.001* NPS vs. HC: *p* = 0.11 PS vs. NPS: *p* = 0.12		*p* = 0.08
Cadence (footfalls/min)	Interaction: Condition x Group *F*(6,393) = 4.7, *p* < 0.001, *p*_*corr*_ < 0.001* Main effects: Group: *F*(2,129) = 21.4, *p* < 0.001* Condition*: F*(3,393) = 610, *p* < 0.001* Covariates: Age: *F*(1,129) = 2.0, *p* = 0.16 BMI: *F*(1,129) = 0.1, *p* = 0.72	Self_Speed	HC: 113 (9.6) NPS: 109 (8.0) PS: 103 (11.8)	PS vs. HC: *p* = 0.005* NPS vs. HC: *p* = 0.67 PS vs. NPS: *p* = 0.24	Interaction: Condition x Groups *F*(3,270) = 4.3, *p* = 0.006, *p*_*corr*_ = 0.010* Main effects: Groups: *F*(1,87) = 10.9, *p* = 0.001* Condition*: F*(3,270) = 418, *p* < 0.001* Covariates: Age: *F*(1,87) = 0.6, *p* = 0.45 BMI: *F*(1,87) = 0.2, *p* = 0.67 Medication: *F*(1,87) = 4.3, *p* = 0.042*	*p* = 0.16
		Max_Speed	HC: 168 (22.2) NPS: 163 (22.0) PS: 145 (19.4)	PS vs. HC: *p* < 0.001* NPS vs. HC: *p* = 0.43 PS vs. NPS: *p* < 0.001*		*p* < 0.001*
		Head_Rec	HC: 109 (14.0) NPS: 106 (16.5) PS: 101 (15.7)	PS vs. HC: *p* = 0.024* NPS vs. HC: *p* = 0.78 PS vs. NPS: *p* = 0.34		*p* = 0.23
		Eyes_Closed	HC: 107 (13.1) NPS: 101 (11.6) PS: 92.8 (13.6)	PS vs. HC: *p* < 0.001* NPS vs. HC: *p* = 0.36 PS vs. NPS: *p* = 0.06		*p* = 0.04*
Stride Length (cm)	Interaction: Condition x Group *F*(6,393) = 0.2, *p* = 0.96, *p*_*corr*_ = 0.96 Main effects: Group: *F*(2,129) = 16.2, *p* < 0.001* Condition*: F*(3,393) = 645, *p* < 0.001* Covariates: Age: *F*(1,129) = 9.9, *p* = 0.002* BMI: *F*(1,129) = 5.7, *p* = 0.019*	Self_Speed	HC: 135 (13.3) NPS: 128 (11.5) PS: 121 (15.6)	PS vs. HC: *p* < 0.001* NPS vs. HC: *p* = 0.28 PS vs. NPS: *p* = 0.27	Interaction: Condition x Groups *F*(3,270) = 0.2, *p* = 0.90, *p*_*corr*_ = 0.96 Main effects: Groups: *F*(1,87) = 4.7, *p* = 0.034* Condition*: F*(3,270) = 441, *p* < 0.001* Covariates: Age: *F*(1,87) = 10.7, *p* = 0.002* BMI: *F*(1,87) = 4.5, *p* = 0.037* Medication: *F*(1,87) = 0.002, *p* = 0.96	*p* = 0.16
		Max_Speed	HC: 171 (19.5) NPS: 165 (24.2) PS: 156 (20.0)	PS vs. HC: *p* < 0.001* NPS vs. HC: *p* = 0.32 PS vs. NPS: *p* = 0.14		*p* = 0.08
		Head_Rec	HC: 125 (15.4) NPS: 117 (18.8) PS: 111 (18.9)	PS vs. HC: *p* < 0.001* NPS vs. HC: *p* = 0.20 PS vs. NPS: *p* = 0.46		*p* = 0.28
		Eyes_Closed	HC: 109 (17.6) NPS: 99.7 (17.5) PS: 91.8 (18.6)	PS vs. HC: *p* < 0.001* NPS vs. HC: *p* = 0.12 PS vs. NPS: *p* = 0.21		*p* = 0.12
FAP	Interaction: Condition x Group *F*(4,262) = 7.2, *p* < 0.001, *p*_*corr*_ < 0.001* Main effects: Group: *F*(2,129) = 5.6, *p* = 0.006* Condition*: F*(2,262) = 60.3, *p* < 0.001* Covariates: Age: *F*(1,129) = 1.8, *p* = 0.18 BMI: *F*(1,129) = 0.5, *p* = 0.48	Self_Speed	HC: 93.1 (10.1) NPS: 97.7 (2.5) PS: 92.0 (13.7)	PS vs. HC: *p* = 0.92 NPS vs. HC: *p* = 0.37 PS vs. NPS: *p* = 0.18	Interaction: Condition x Groups *F*(2,180) = 1.8, *p* = 0.18, *p*_*corr*_ = 0.21 Main effects: Groups: *F*(1,87) = 6.1, *p* = 0.016* Condition*: F*(2,180) = 64.3, *p* < 0.001* Covariates: Age: *F*(1,87) = 1.6, *p* = 0.21 BMI: *F*(1,87) = 0.9, *p* = 0.35 Medication: *F*(1,87) = 2.2, *p* = 0.14	*p* = 0.14
		Max_Speed	—	—		-
		Head_Rec	HC: 92.7 (9.99) NPS: 92.9 (6.8) PS: 87.1 (14.8)	PS vs. HC: *p* = 0.07 NPS vs. HC: *p* = 1.0 PS vs. NPS: *p* = 0.16		*p* = 0.13
		Eyes_Closed	HC: 90.2 (10.7) NPS: 88.8 (10.1) PS: 79.0 (15.3)	PS vs. HC: *p* < 0.001* NPS vs. HC: *p* = 0.90 PS vs. NPS: *p* = 0.006*		*p* = 0.007*
Variance in stride time (%CV)	Interaction: Condition x Group *F*(6,393) = 3.8, *p* = 0.001, *p*_*corr*_ = 0.004* Main effects: Group: *F*(2,129) = 5.2, *p* = 0.007* Condition*: F*(3,393) = 85.4, *p* < 0.001* Covariates: Age: *F*(1,129) = 4.1, *p* = 0.044* BMI: *F*(1,129) = 0.03, *p* = 0.87	Self_Speed	HC: 1.91 (1.20) NPS: 1.88 (0.89) PS: 2.37 (1.29)	PS vs. HC: *p* = 0.38 NPS vs. HC: *p* = 1.0 PS vs. NPS: *p* = 0.58	Interaction: Condition x Groups *F*(3,270) = 6.5, *p* < 0.001, *p*_*corr*_ = 0.002* Main effects: Groups: *F*(1,87) = 3.5, *p* = 0.063* Condition*: F*(3,270) = 62.7, *p* < 0.001* Covariates: Age: *F*(1,87) = 2.5, *p* = 0.11 BMI: *F*(1,87) = 0.02, *p* = 0.89 Medication: *F*(1,87) = 2.4, *p* = 0.12	*p* = 0.41
		Max_Speed	HC: 3.45 (1.60) NPS: 4.28 (2.35) PS: 3.42 (1.66)	PS vs. HC: *p* = 1.0 NPS vs. HC: *p* = 0.16 PS vs. NPS: *p* = 0.11		*p* = 0.041*
		Head_Rec	HC: 2.93 (1.03) NPS: 2.88 (1.35) PS: 4.04 (2.35)	PS vs. HC: *p* = 0.006* NPS vs. HC: *p* = 1.0 PS vs. NPS: *p* = 0.036*		*p* = 0.026*
		Eyes_Closed	HC: 4.85 (2.45) NPS: 4.47 (1.28) PS: 5.94 (2.45)	PS vs. HC: *p* = 0.007* NPS vs. HC: *p* = 0.78 PS vs. NPS: *p* = 0.005*		*p* = 0.004*
Variance in stride length (%CV)	Interaction: Condition x Group *F*(6,393) = 2.9, *p* < 0.001, *p*_*corr*_ = 0.014* Main effects: Group: *F*(2,129) = 7.1, *p* = 0.001* Condition*: F*(3,393) = 290.6, *p* < 0.001* Covariates: Age: *F*(1,129) = 2.5, *p* = 0.12 BMI: *F*(1,129) = 2.3, *p* = 0.13	Self_Speed	HC: 1.86 (0.61) NPS: 2.0 (0.69) PS: 2.44 (1.37)	PS vs. HC: *p* = 0.45 NPS vs. HC: *p* = 0.98 PS vs. NPS: *p* = 0.73	Interaction: Condition x Groups *F*(3,270) = 2.3, *p* = 0.08, *p*_*corr*_ = 0.11 Main effects: Groups: *F*(1,87) = 4.5, *p* = 0.037* Condition*: F*(3,270) = 208.1, *p* < 0.001* Covariates: Age: *F*(1,87) = 1.7, *p* = 0.20 BMI: *F*(1,87) = 2.6, *p* = 0.11 Medication: *F*(1,87) = 1.2, *p* = 0.28	*p* = 0.54
		Max_Speed	HC: 2.34 (0.78) NPS: 2.58 (1.0) PS: 2.38 (1.10)	PS vs. HC: *p* = 1.0 NPS vs. HC: *p* = 0.92 PS vs. NPS: *p* = 0.90		*p* = 0.62
		Head_Rec	HC: 3.78 (1.71) NPS: 3.57 (1.35) PS: 4.65 (2.48)	PS vs. HC: *p* = 0.15 NPS vs. HC: *p* = 0.92 PS vs. NPS: *p* = 0.14		*p* = 0.09
		Eyes_Closed	HC: 7.61 (3.20) NPS: 8.09 (2.89) PS: 9.74 (4.11)	PS vs. HC: *p* < 0.001* NPS vs. HC: *p* = 0.72 PS vs. NPS: *p* = 0.010*		*p* = 0.009*

Main ANCOVA: analysing the effect of three groups and four conditions on gait parameter (except FAP with three conditions) controlling for age and BMI. ANCOVA patients: analysing the effect of only patient groups (NPS vs. PS) and four conditions on gait parameter (except FAP with three conditions) controlling for age, BMI, and medication. Contrasts are Tukey corrected for multiple comparison within each model. Additionally, the *p*-values of the interaction effects of the 12 ANCOVA models are FDR corrected for multiple comparison (*p*_*corr*_).

*BMI* Body Mass Index, *sd* standard deviation, *PS* psychomotor slow, *NPS* non-psychomotor slow, *HC* healthy controls, *Self_Speed* self-selected speed, *Max_Speed* maximum speed, *Head_Rec* head reclination, *Eyes_Closed* eyes closed, *vs.* versus, *FAP* functional ambulation performance score, *%CV* %-coefficient of variance.

*p and/or *p*_*corr*_ ≤ 0.05.

When comparing the two patient groups separately correcting for age, BMI, and current medication, the NPS had higher velocity, cadence, and lower variance in stride length and time than the PS in the Max_Speed condition. During the condition Eyes_Closed, the NPS differed significantly from the PS with a higher cadence and FAP, and lower variance in stride length and time. The variance in stride time was also significantly lower in the NPS during the Head_Rec condition. Stride length indicated no group difference in any condition. Also, in the Self_Speed condition, we did not find significant group differences for any parameter. Please note, that the classification of underweight, normal-weight, overweight, and obese people was similar across the three groups (Supplementary Table S2) and that there were no triple interactions between groups, conditions, and sex for any of the gait parameters (Supplementary Table S3).

### Correlations

**Fig. 2:**
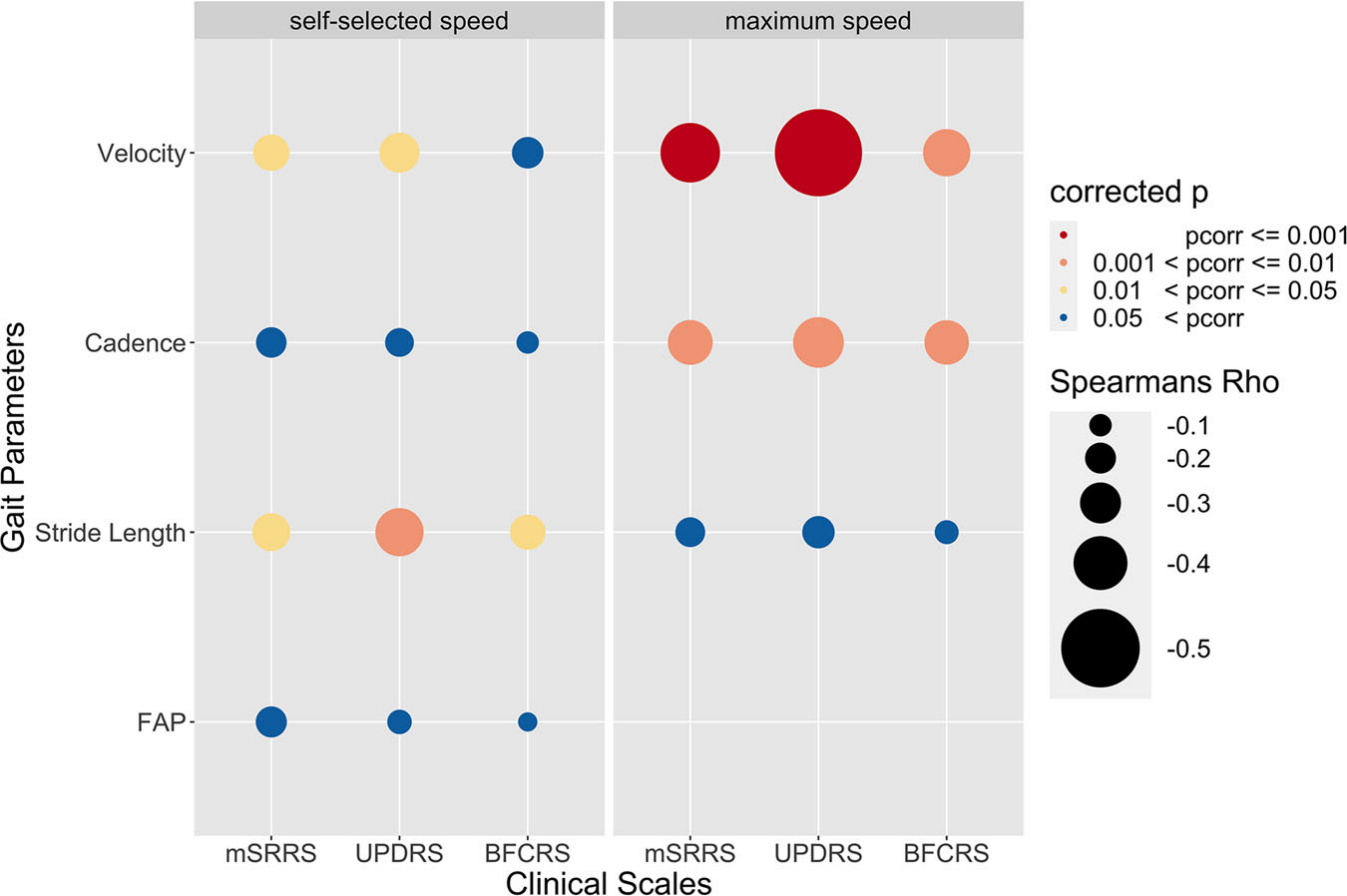
Correlation plot between gait parameters and clinical scales across all patients (*N* = 92). The size of the dots represents the Spearman correlation coefficient rho; the colour of the dots represents *p*_*corr*_. For exact rho and *p*-values see Supplementary Table S8. For raw association data see Supplementary Figs. S2 and S3. N number of participants, mSRRS motoric part of the Salpêtrière Retardation Rating Scale, BFCRS Bush-Francis Catatonia Rating Scale, UPDRS Unified Parkinson Disease Rating Scale Part III, *p*_*corr*_ FDR corrected *p*-values for multiple comparisons.

Based on the Spearman correlation analysis (Fig. 2, Supplementary Figs. S2 and S3, Supplementary Table S4), patients with more severe motor abnormalities (psychomotor slowing, catatonia, and parkinsonism) had slower velocity (Self_Speed and Max_Speed), lower cadence (Max_Speed), and shorter stride length (Self_Speed). Correlation coefficients were generally stronger in Max_Speed than in Self_Speed, except for stride length, which correlated the strongest with UPDRS in Self_Speed.

Additionally, we tested the correlation between Self_Speed or Max_Speed with the total BNSS plus two subdomains (Supplementary Table S5). The BNSS total score was negatively correlated with gait parameters (velocity, cadence, stride length only in Self_Speed), as was the subdomain avolition (velocity, cadence only in Self_Speed, stride length only in Self_Speed), while the subdomain expressivity only correlated with Max_Speed of velocity and cadence.

## Discussion

This study provides a unique evaluation of gait in schizophrenia comparing patients with and without psychomotor slowing to healthy controls. First, our large cohort of patients with schizophrenia (*N* = 92) corroborated previous findings of slower gait velocity compared to healthy controls. Moreover, we observed aberrant gait profiles in patients across multiple parameters such as lower cadence, shorter stride length, poorer gait performance, and less regular gait in several conditions. Especially, we observed most pronounced gait impairments in patients with psychomotor slowing according to expert ratings, who failed to adapt during Eyes_Closed or Max_Speed conditions. The patients without psychomotor slowing presented an intermediate gait profile between healthy subjects and patients with psychomotor slowing, supporting a dimensional view of motor behaviour and gait.

The Self_Speed condition is an ecological walking situation reflecting spontaneous gait profiles in daily life. In this specific condition, patients with psychomotor slowing had slower velocity, lower cadence, and shorter stride length compared to controls. However, the gait profiles of patients without psychomotor slowing were similar to healthy controls.

Prior research indicated a specific pattern of gait impairments in schizophrenia, including disturbed stride length regulation at unchanged cadence, resulting in reduced velocity^19,25,38^. In line with previous studies, we found slower velocity in patients in comparison to healthy controls. In contrast, only our study and Presta et al. noted lower cadence in patients^32^. This study is the first to evaluate FAP in patients with schizophrenia. FAP is considered a useful measure to characterise gait profiles in other illnesses such as Parkinson’s disease^39^, multiple sclerosis^40^, and muscular dystrophy^41^. Here, we found no group difference in self-selected walking speed, challenging the sensitivity of FAP to gait impairments in schizophrenia with or without psychomotor slowing.

In addition to normal gait, we tested gait in complex situations: during speedy walking or visual restriction, e.g. checking mobile devices. When asked to walk faster, all participants were able to increase their walking speed, but patients with psychomotor slowing were unable to speed up to the same level as controls. This is why, in the challenging Max_Speed condition there is a significant difference between all three groups, in contrast to Self_Speed where only healthy controls and patients with psychomotor slowing differ. This finding indicates that patients with psychomotor slowing are less capable to adapt to challenging walking tasks compared to patients without psychomotor slowing or healthy controls. The same pattern emerged in the other challenging conditions (e.g. Eyes_Closed): with increasing task difficulty, all participants decreased velocity, cadence, and stride length; however, decreases were most prominent in patients with psychomotor slowing.

Conditions other than spontaneous speed are less commonly investigated in the literature. In line with our results, Akbaş et al. reported reduced velocity during maximum walking speed in patients with schizophrenia compared to healthy controls^28^. However, during the 6 min walking task at maximum velocity, both the healthy controls and patients were much slower than in the Max_Speed condition of our study. For conditions with closed eyes, the data is inconsistent. Using an ataxia battery, one study found a group difference in gait performance between healthy controls and patients^42^, while others failed to find such differences in velocity, cadence, and stride length during tandem gait^29^. Our finding of a generalised disturbance, that is more pronounced in complex gait conditions, argues for a deficit in central motor control in individuals with schizophrenia and psychomotor slowing^4,43^.

We correlated the gait parameters of all patients during Self_Speed and Max_Speed with expert ratings of psychomotor slowing, parkinsonism, and catatonia to test for an association between gait performance and severity of hypokinetic motor abnormalities. Gait abnormalities were more strongly associated with psychomotor slowing and parkinsonism than with catatonia. Velocity is affected by hypokinetic movement disorders during both Self_Speed and Max_Speed. Cadence might be more robust than stride length, as cadence is not associated with hypokinesia during Self_Speed whereas stride length is. During the more stressful Max_Speed, the disturbance in cadence then overrides the stride length impairment.

Previous research has rarely looked at the association between expert ratings of motor abnormalities and objective measures of gait. In line with our results, Martin et al. reported an association of the Heidelberg Neurological Soft Signs scale with velocity and stride length during self-selected speed^19^. Likewise, worse or slowed gait performance was noted in schizophrenia and associated with hypokinetic movement disorders such as parkinsonism^28,29^.

Aberrant gait might be a result of the well-known motor system dysfunction in schizophrenia. Motor abnormalities have been linked to abnormal brain structure^44,45^ and connectivity^46^ within the motor network, including the cerebellum^47^. Aberrant functional connectivity in the motor system in schizophrenia^46,48^ correlated with behavioural motor abnormalities, suggesting that the motor system in schizophrenia might be out of balance^46,48,49^. Especially, due to its key role in the planning, execution, coordination, and control of gait^50^, cerebellar alterations might lead to severe gait abnormalities across various psychiatric and neurological conditions^51^. Moreover, functional brain alterations in catatonia include hyperconnectivity from the precentral gyrus to cerebellar areas and the basal ganglia^52^ as well as higher perfusion of the supplementary motor area (SMA)^53,54^.

In sum, these results suggest that restoring the balance in the sensorimotor network might alleviate the motor abnormalities in schizophrenia and potentially gait parameters. Non-invasive brain stimulation, particularly repetitive transcranial magnetic stimulation (rTMS) holds great promise for the treatment of altered motor function in schizophrenia^55^. A recent randomised, double-blind pilot study demonstrated that inhibitory rTMS over the SMA could improve psychomotor slowing^56^. Other potential treatment options include physical exercise, such as Movement or Sports Therapy. Physical exercise has shown to be effective in improving quality of life, depressive symptoms, cognition in multiple psychiatric disorders^57,58^.

Negative symptom severity may also affect motor behaviour including gait parameters^7,22,59–62^. Previous reports failed to demonstrate a consistent association between negative symptoms and gait parameters; however, negative symptoms were only assessed with PANSS negative instead of a dedicated scale^19,29,30^. In our study, we tested the association of gait during Self_Speed and Max_Speed with the total BNSS and the subdomains Avolition and Expressivity. More pronounced negative symptoms were linked to decreased velocity, cadence, and stride length. Slower velocity was associated with negative symptoms at both Self_Speed and Max_Speed. Patients with avolition were mostly impaired during Self_Speed, whereas patients suffering from reduced expressivity show impairment only during Max_Speed.

The strengths of this study are a detailed objective assessment of gait in a large sample of patients with schizophrenia under various conditions, focussing specifically on patients with psychomotor slowing. However, the results have to be considered in light of some limitations. First, the sample size of the patients without psychomotor slowing is rather small, perhaps hampering the detection of smaller differences between patient groups. In addition, current and past medication might have impacted gait performance. For example, Putzhammer et al. found that patients on conventional antipsychotics had slower gait velocity and shorter stride length compared to drug-naïve and atypically treated patients, who differed from healthy controls^18^. While we carefully controlled all the analyses for the current dosage of antipsychotic medication, we could not control for cumulated lifetime antipsychotic exposure. Also, as height and weight could influence the gait measurements, using them as matching variables would have provided a better between-groups comparison. However, in the present study, we took this information into account by adding BMI as a controlling variable. Moreover, the classification of underweight, normal-weight, overweight, and obese people was similar across the three groups. On top of that, the present study included participants who have a relatively low BMI. The socio-economic situation of Switzerland might have influenced this distribution and might make the current results not replicable in populations with higher BMIs. Future studies including a larger BMI range are needed to address this specific question. Finally, we did not measure or control the level of motivation and attention of the participants. While we always supported patients to ensure optimal performance, we cannot neglect the potential impact of negative and cognitive symptoms that might reduce patients’ motivation and increase attentional deficits^63,64^.

## Conclusion

Psychomotor slowing in schizophrenia spectrum disorders is associated with clearly impaired gait profiles. While patients without psychomotor slowing had some alterations in their gait, they still presented a similar gait profile as healthy controls. Our study suggests that gait impairments follow a continuum with the healthy population at one end and patients with psychomotor slowing on the other end, and patients without psychomotor slowing in the intermediate position. Particularly, velocity at self-selected speed could be a simple, readily available, and accurate method to assess psychomotor slowing in clinical routine. However, more challenging gait conditions, such as maximum speed, inform on hypokinetic movement disorders and negative symptom severity. Finally, gait abnormalities may indicate aberrant neural activity in the motor circuit in schizophrenia.

## Material and methods

### Participants

We included baseline data of 70 patients with schizophrenia spectrum disorders according to the Structured Clinical Interview for DSM-5® (SCID-5), from the ongoing OCoPS-P study (Overcoming Psychomotor Slowing in Psychosis; ClinicalTrials.gov Identifier: NCT03921450). Patients with psychomotor slowing (PS) were included if they had a total score of ≥15 on the Salpêtrière Retardation Rating Scale^33^ (SRRS). In addition, we recruited a group of 22 patients without psychomotor slowing (NPS) (total score < 15). Further, we included 42 age and sex-matched healthy controls (HC) (Table 1). Exclusion criteria were age <18 or >60 years, active substance dependence (except nicotine), history of neurological disorders, history of traumatic brain injury, epilepsy, claustrophobia, metal implants, hearing problems, and tinnitus. Additionally, healthy participants were excluded for lifetime psychiatric illness or for having first-degree relatives with psychosis.

Recruitment of patients took place at the in- and outpatient departments of the University Hospital of Psychiatry and Psychotherapy in Bern, Switzerland. HC were recruited via flyers and online advertisements. All participants were informed and signed the consent form before entering the study. The study protocol adhered to the Declaration of Helsinki (World Medical Association, 2013) and was approved by the local ethics committee (KEK-BE 2018-02164).

### Procedures

A higher score on any of the general psychopathology scales and motor rating scales indicates a higher symptom severity or stronger motor impairment, respectively. Participants were assessed by psychiatrists who were trained by the last author to achieve high interrater reliability (kappa > 0.8). All assessments were conducted on the same day.

#### General psychopathology

We assessed the overall symptom severity using the Positive and Negative Syndrome Scale^34^ (PANSS). Mean olanzapine equivalents (OLZ eq.) were calculated according to Leucht et al.^65^. With the Brief Negative Symptom Scale^35^ (BNSS) we assessed five domains of negative symptoms (anhedonia, asociality, avolition, emotional expressivity, and alogia).

#### Motor rating scales

Psychomotor slowing was assessed using the SRRS^33^ that has been applied across depression and the broad psychosis spectrum^10,24,66,67^. As the SRRS comprises items on motor behaviour, cognitive impairment, and depressive symptoms, researchers have created a subscore solely comprised of motor items, the motor-SRRS (mSRRS^10^). The mSRRS summarises the first five psychomotor items (e.g. slowed gait, slowed movement of limbs or trunk, reduced head or gaze movement, reduced facial expression, slowed speech, and monotonous voice) and the last item (appreciation of general retardation)^10^. The Unified Parkinson Disease Rating Scale^36^ (UPDRS) part III was applied to assess parkinsonism. The Bush-Francis Catatonia Rating Scale^37^ (BFCRS) was used to quantify catatonia severity.

#### Instrumental measures

We evaluated participants’ gait using the GAITRite® system (platinum GAITRite walkway, CIR Systems Inc., Sparta, NJ 07871; USA). It consists of a 0.89 m wide and 7.01 m long electronic walkway with 20,040 sensors, that capture clinical information on participants’ gait. The walkway looks like a grey carpet. For each condition, participants were asked to walk on the walkway four times to calculate the average walking parameters.

##### Gait conditions

The gait conditions can be categorised into ecological conditions, (i) walking at comfortable self-selected speed (Self_Speed), (ii) walking as fast as possible without running while the investigator cheered to boost participants’ performance (Max_Speed), and distractor conditions (iii) walking while tilting their head back and only look at the ceiling (Head_Rec), (iv) walking while having eyes closed (Eyes_Closed).

##### Gait parameters

We evaluated the following six gait parameters: (i) velocity (cm/s) i.e. walked distance (cm) by time (s), (ii) cadence (footfalls/min) i.e. number of steps taken per minute, (iii) stride length (cm) i.e. the distance between the heel points of two consecutive footfalls of the same foot, and (iv) the functional ambulation performance (FAP) score which is a quantitative measure that objectively assesses gait performance in adults using selected time and distance parameters as well as the dynamic base of support^39^. From a maximum of 100, points are deducted for deviating from the norm^68^. The FAP is a sensible measure during comfortable unhurried gait, therefore it was not calculated for Max_Speed^39,68^. (v–vi) Additionally, we included measures of variance for the stride time (time between two subsequent footfalls of the same foot) (s) and the stride length (cm). The %-coefficient of variance (%CV) is calculated by dividing the standard deviation by the mean and multiplying by 100.

### Statistical analyses

All analyses were computed with RStudio (version 1.1.463) and R (version 4.2.0). First, we tested for differences in the demographic data between HC, NPS, and PS using an ANOVA. To test for equal distribution of sex in our data, we applied a chi-square test. Nonparametric Mann–Whitney U-tests were computed to test for differences in clinical scales between PS and NPS. Additionally, we tested between-sex differences in clinical variables within the groups using Mann–Whitney U-tests (Supplementary Table S1).

Next, for each of the six gait parameters, we used a repeated-measures ANCOVA with group (HC, NPS, PS) and condition (Self_Speed, Max_Speed, Head_Rec, Eyes_Closed) as factors and age and BMI as controlling variables (Fig. 1, Table 2, raw data in Supplementary Fig. S1). In addition to adding BMI as a controlling variable, we checked that the classification of underweight, normal-weight, overweight, and obese people was similar across the three groups. (Supplementary Table S2). To explicitly look at differences between HC and the two patient groups during specific conditions, we performed post hoc *t*-tests with Tukey correction for multiple comparisons. Additionally, to account for medication effects, a dedicated ANCOVA was performed between the two patient groups with OLZ eq. as an additional covariate. Due to the large number of comparisons performed, we performed an additional correction for multiple comparisons on the interaction effect of each model (*N* = 12) using FDR-adjusted p-values (p_corr_).

Additionally, we tested the effect of sex on gait parameters in a supplementary ANCOVA (Supplementary Table S3), as well as the effect of duration of illness and number of episodes in a correlation analysis and a supplementary ANCOVA (Supplementary Table S6 and S7). We also analysed the sole effect of group on gait performance only in self-selected speed (Supplementary Table S8) and the sole effect of condition on gait performance separately per group (Supplementary Table S9).

Nonparametric Spearman partial correlations were calculated for all patients between four gait parameters (velocity, cadence, stride length, FAP) and three clinical scales (mSRRS, UPDRS, BFCRS). Only the conditions Self_Speed and Max_Speed were included in the correlational analyses, except for FAP, where Max_Speed was not considered. The partial correlations were controlled for the covariates age, BMI, and medication. The resulting *p*-values were corrected for multiple comparisons using FDR (*p*_corr_). The raw correlation data are presented in scatterplots in the supplements (Supplementary Figs. S2 and S3).

We also performed an exploratory correlation analysis between the gait parameters and the BNSS total score and two subdomains (Supplementary Table S5). *p*_corr_ < 0.05 was considered significant.

## Supplementary information

Supplementary Material

## Data Availability

The data that support the findings of this study are available from the corresponding author upon reasonable request.
