# Supplementary material for: Psychomotor slowing alters gait velocity, cadence, and stride length and indicates negative symptom severity in psychosis
Source: Schizophrenia (Heidelb). 2022 Dec 30;8(1):116. doi: 10.1038/s41537-022-00324-x (PMC9803648; doi:10.1038/s41537-022-00324-x)
Supplement: Supplementary file 1 — Supplementary Material [file 41537_2022_324_MOESM1_ESM.docx]

**Supplementary Material**

**Psychomotor Slowing alters Gait Velocity, Cadence, and Stride Length and indicates Negative Symptom Severity in Psychosis**

*Melanie G. Nuoffer, MSc*^1,4^; Stephanie Lefebvre, PhD^1^; Niluja Nadesalingam, MSc^1,4^; Danai Alexaki, MD^1,2^; Daniel Baumann Gama, MD^1^; Florian Wüthrich^1,4^, MD; Alexandra Kyrou, MD^1^, Hassen Kerkeni, MD^3^, Roger Kalla, MD PhD^3^, and Sebastian Walther, MD PhD^1^*

**Supplementary Figures**

Figure S1: Raw data with violin border to indicate distribution and density of gait performance per condition and per group

*Note*: Individual values of participants are represented by dots and violin border indicates distribution. Data is separated into 3 groups and 4 walking conditions (except FAP with only 3 conditions). FAP: functional ambulation performance score; blue: healthy controls; yellow: non-psychomotor slow; red: psychomotor slow.

Figure S2: Raw data showing the association between gait parameters and clinical scales during self-selected speed

*Note*: For each patient (22 NPS in blue and 70 PS in red) the raw data of the gait parameters and the clinical scales is indicated with a dot. Grey line indicates the linear association between the two variables across all patients uncontrolled for covariates.

NPS: non-psychomotor slow in yellow; PS: psychomotor slow in red; mSRRS: motoric part of the Salpêtrière Retardation Rating Scale; UPDRS: Unified Parkinson Disease Rating Scale Part III; BFCRS: Bush-Francis Catatonia Rating Scale.

Figure S3: Raw data showing the association between gait parameters and clinical scales during maximum speed

*Note*: For each patient (22 NPS in blue and 70 PS in red) the raw data of the gait parameters and the clinical scales is indicated with a dot. Grey line indicates the linear association between the two variables across all patients uncontrolled for covariates.

NPS: non-psychomotor slow in yellow; PS: psychomotor slow in red; mSRRS: motoric part of the Salpêtrière Retardation Rating Scale; UPDRS: Unified Parkinson Disease Rating Scale Part III; BFCRS: Bush-Francis Catatonia Rating Scale.

Figure S1: Raw data with violin border to indicate distribution of gait performance per condition and per group


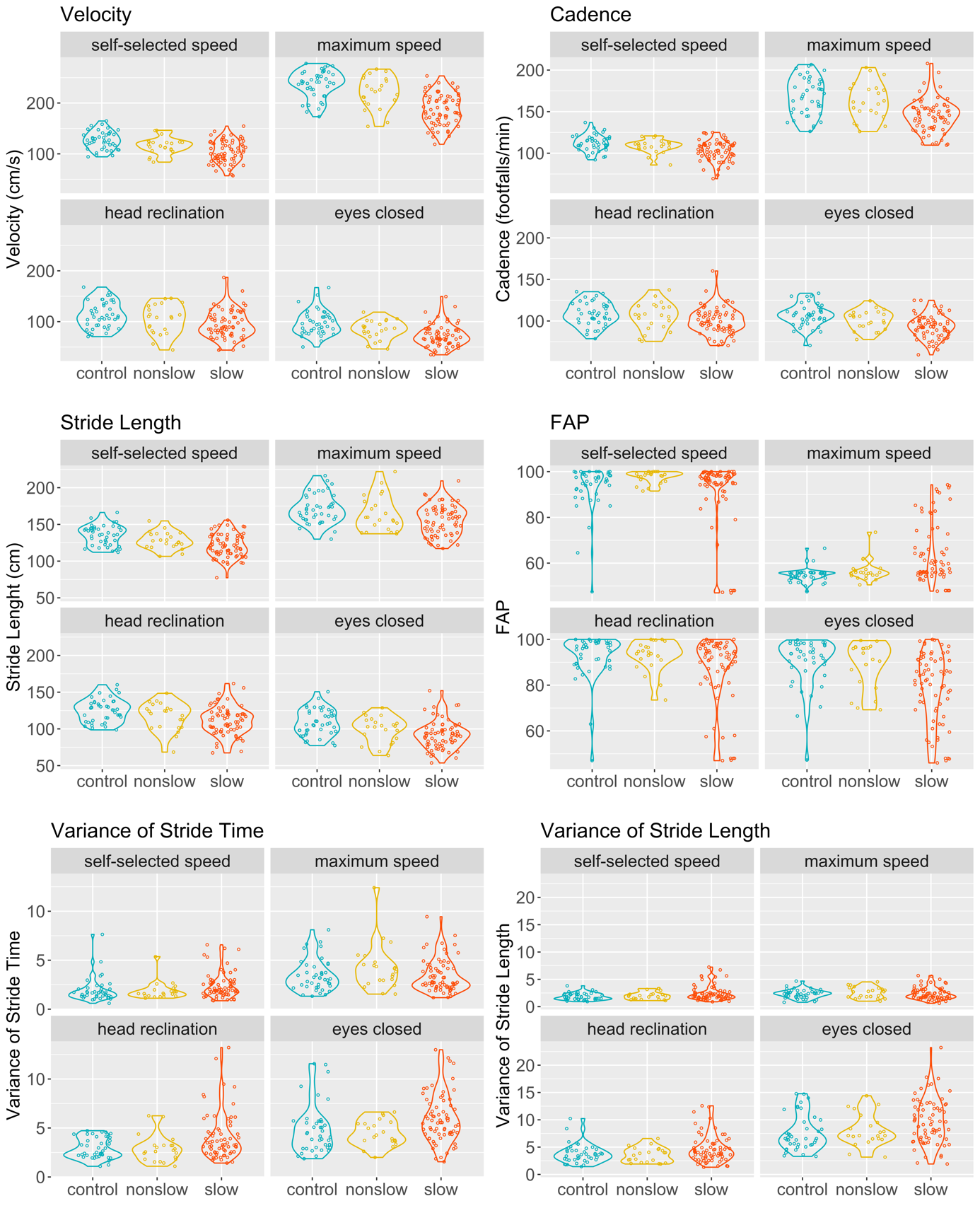


*Note*: Individual values of participants are represented by dots and violin border indicates distribution. Data is separated into 3 groups and 4 walking conditions (except FAP with only 3 conditions). FAP: functional ambulation performance score; blue: healthy controls; yellow: non-psychomotor slow; red: psychomotor slow.

Figure S2: Raw data showing the association between gait parameters and clinical scales during self-selected speed


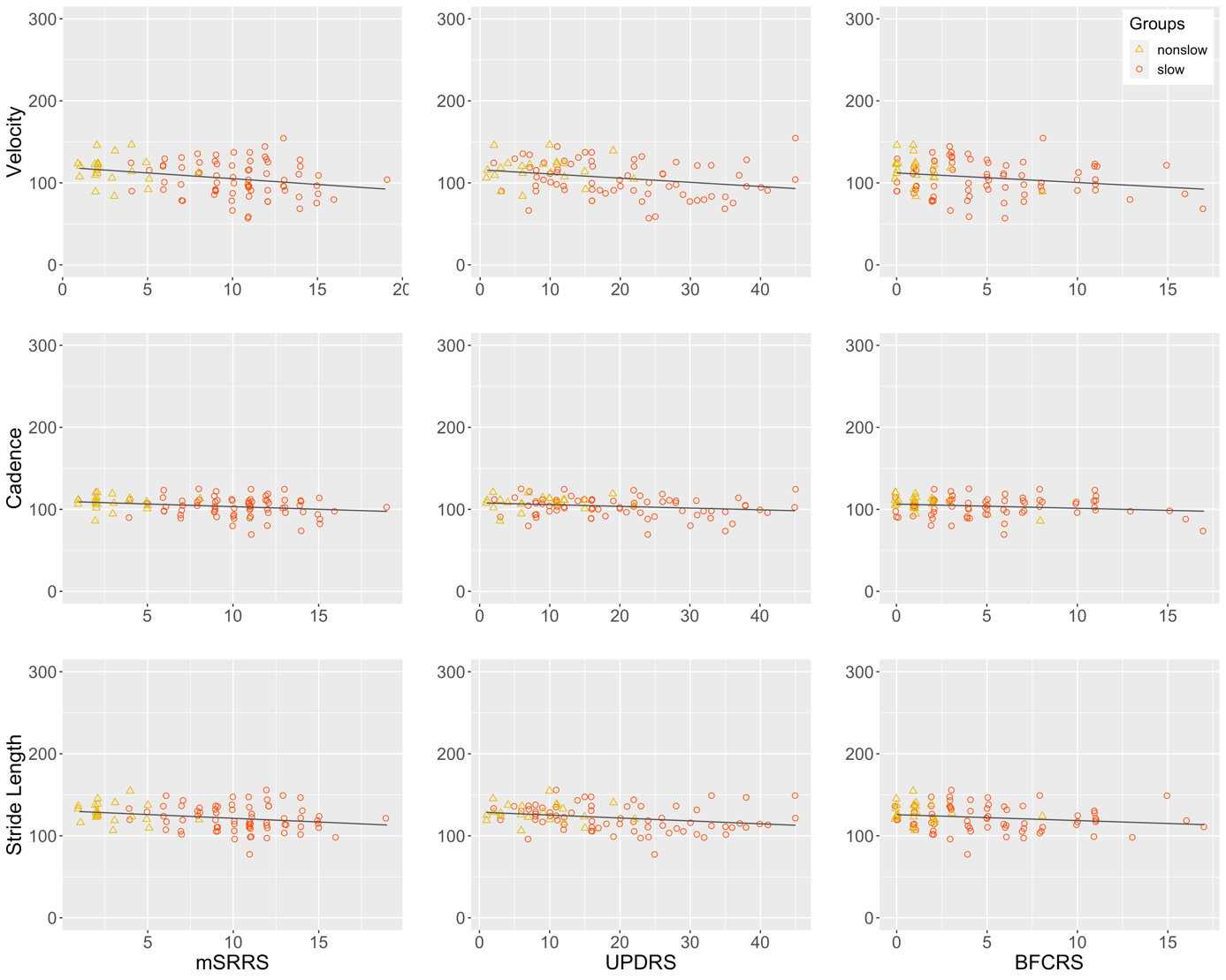


*Note*: For each patient (22 NPS and 70 PS) the raw data of the gait parameters and the clinical scales is indicated with a dot. Grey line indicates the linear association between the two variables across all patients uncontrolled for covariates.

yellow triangles: non-psychomotor slow (NPS); red circles: psychomotor slow (PS); mSRRS: motoric part of the Salpêtrière Retardation Rating Scale; UPDRS: Unified Parkinson Disease Rating Scale Part III; BFCRS: Bush-Francis Catatonia Rating Scale.

Figure S3: Raw data showing the association between gait parameters and clinical scales during maximum speed


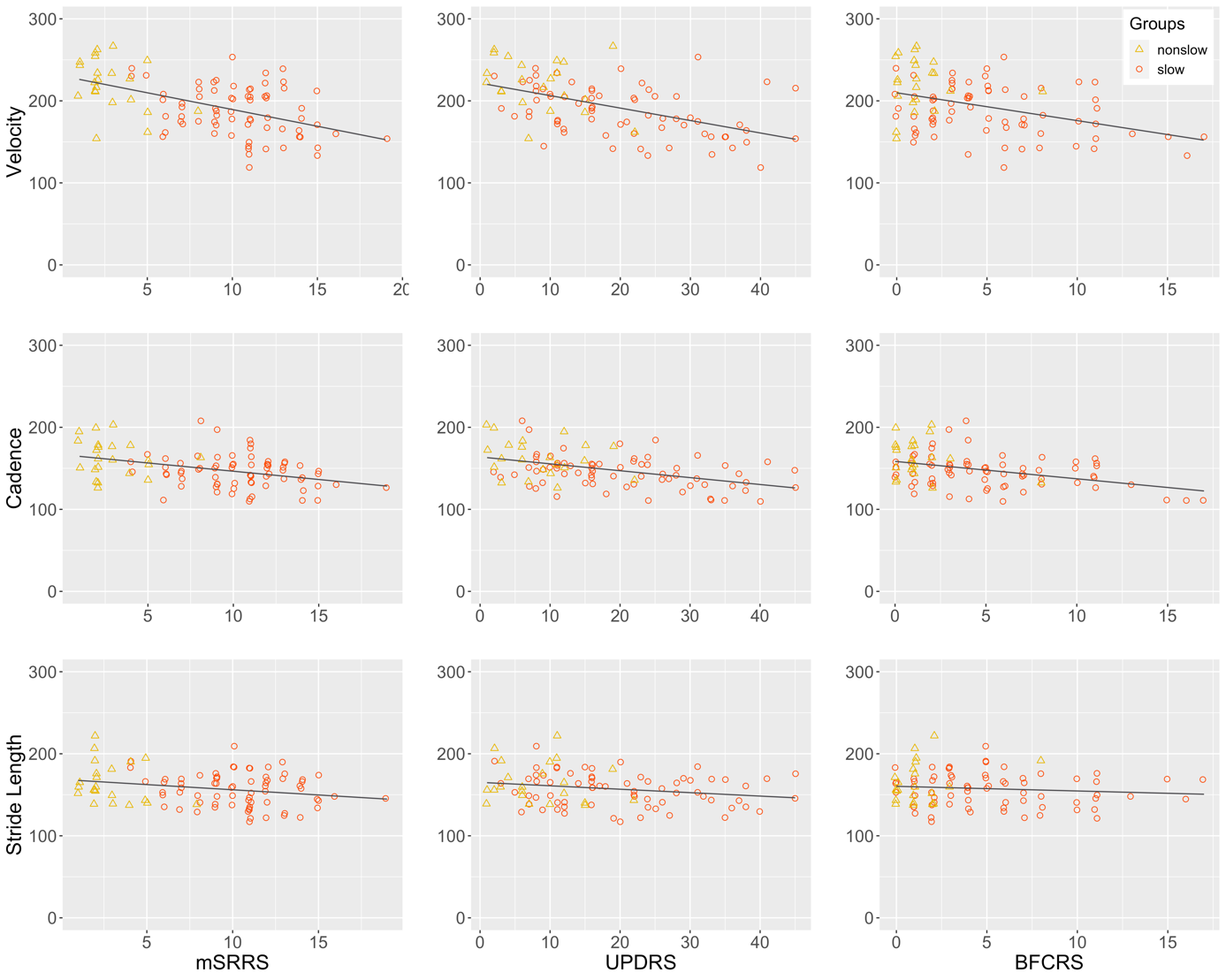


*Note*: For each patient (22 NPS and 70 PS) the raw data of the gait parameters and the clinical scales is indicated with a dot. Grey line indicates the linear association between the two variables across all patients uncontrolled for covariates.

yellow triangles: non-psychomotor slow (NPS); red circles: psychomotor slow (PS); mSRRS: motoric part of the Salpêtrière Retardation Rating Scale; UPDRS: Unified Parkinson Disease Rating Scale Part III; BFCRS: Bush-Francis Catatonia Rating Scale.

**Supplementary Tables**

Table S1: Comparison of Demographics, Clinical Scales, and Gait Parameters between Sexes during self-selected Speed

*Note*: For each measure the mean is indicated with the standard deviation (SD) in brackets separately for female and male participants. For comparison between sexes a Mann-Whitney-U-Test is performed (W). P-values are uncorrected.

N: number of participants; f: female; m: male; BMI: Body Mass Index; OLZ eq.: Olanzapine-equivalent (mg/day); PANSS: Positive And Negative Syndrome Scale; neg.: negative; pos.: positive; SRRS: Salpêtrière Retardation Rating Scale; mSRRS: motoric part of the Salpêtrière Retardation Rating Scale; BFCRS: Bush-Francis Catatonia Rating Scale; UPDRS: Unified Parkinson Disease Rating Scale Part III; BNSS: Brief Negative Symptom Scale. FAP: functional ambulation performance score.

** p* ≤ .05.

Table S2: Test for equal distribution of BMI categories between groups

*Note*: For each cell, the number of participants is indicated. For comparison between groups a Fisher’s exact test for count data is performed (alternative = two-sided).

BMI: Body Mass Index; HC: healthy controls; NPS: non-psychomotor slow; PS: psychomotor slow.

** p* ≤ .05.

Table S3: Effects of Group, Condition, and Sex on Gait Performance for each Gait Parameter

*Note*: For each comparison (btw. 3 groups, 4 conditions, 2 genders) an ANCOVA is performed, controlled for age and BMI. For FAP there are only 3 conditions included. P-values are uncorrected for multiple comparison.

f: female; m: male; BMI: Body Mass Index; FAP: functional ambulation performance score.

** p* ≤ .05.

Table S4: Spearman Correlations between Gait Parameters and Clinical Scales across all Schizophrenia Patients (N = 92) controlled for Age, BMI, and Medication

*Note*: Spearman correlations are calculated across all patients (70 PS and 22 NPS) and controlled for age, BMI, and medication (rho). All p-values are FDR corrected for multiple comparison (*p_corr_*).

mSRRS: motoric part of the Salpêtrière Retardation Rating Scale; UPDRS: Unified Parkinson Disease Rating Scale Part III; BFCRS: Bush-Francis Catatonia Rating Scale; Self_Speed: self-selected speed; Max_Speed: maximum speed; FAP: functional ambulation performance score.

*** *p_corr_* ≤ .05

Table S5: Spearman Correlations between Gait Parameters and BNSS plus Subdomains across all Schizophrenia Patients (N = 92)

*Note*: Spearman correlations are calculated across all patients (70 PS and 22 NPS) and controlled for age, BMI, and medication (rho). As this analysis is exploratory, p-values are uncorrected for multiple comparison.

N = number of participants; BNSS: Brief Negative Symptom Scale; Self_Speed: self-selected speed; Max_Speed: maximum speed; FAP: functional ambulation performance score.

** p* ≤ .05.

Table S6: Correlation of number of episodes and duration of illness with gait parameters during self-selected speed

*Note*: Spearman correlations are calculated across all patients (70 PS and 22 NPS) (rho). As this analysis is exploratory, p-values are uncorrected for multiple comparison. NPS: non-psychomotor slow; PS: psychomotor slow.

** p* ≤ .05.

Table S7: Effects of duration of illness and number of episodes on the ANCOVA patients

*Note*: ANCOVA patients: analysing the effect of only patient groups (NPS vs. PS) and 4 conditions on gait controlling for age, BMI, and medication. Additional ANCOVA patients: adding the covariates number of episodes and duration of illness in the previous ANCOVA.

BMI: Body Mass Index; sd: standard deviation; PS: psychomotor slow; NPS: non-psychomotor slow; HC: healthy controls.

** p* ≤ .05

Table S8: Effect of Group on Gait Performance for each Gait Parameter during self-selected Speed

*Note*: For each comparison (btw. 3 groups) an ANCOVA is performed, controlled for age and BMI. P-values are uncorrected for multiple comparison. For each measure the mean is indicated with the standard deviation (SD) in brackets. Contrasts are automatically Tukey corrected for multiple comparison within each model.

BMI: Body Mass Index; PS: psychomotor slow; NPS: non-psychomotor slow; HC: healthy controls.

** p* ≤ .05.

Table S9: Effect of Condition on Gait Performance for each Gait parameter separately for each Group

*Note*: A) For each comparison (btw. 4 condition) an ANCOVA is performed, controlled for age and BMI. P-values are uncorrected for multiple comparison. For each measure the mean is indicated with the standard deviation (SD) in brackets.

B) Contrasts are automatically Tukey corrected for multiple comparison within each model.

BMI: Body Mass Index; HC: healthy controls; NPS: non-psychomotor slow; PS: psychomotor slow; N: number of participants; Self_Speed: self-selected speed; Max_Speed: maximum speed; Head_Rec: head reclination; Eyes_Closed: eyes closed; Diff: difference.

Table S1: Comparison of Demographics, Clinical Scales, and Gait Parameters between Sexes during self-selected Speed

|  | Healthy controls (HC) | | Nonslow (NPS) | | Psychomotor slow (PS) | |
| --- | --- | --- | --- | --- | --- | --- |
|  | *N* = 42 (50% female) | | *N* = 22 (55% female) | | *N* = 70 (50% female) | |
| A) Demographics | | | | | | |
|  | mean (SD) | Difference  f vs. m | mean (SD) | Difference  f vs. m | mean (SD) | Difference  f vs. m |
| Age (years) | f: 33 (12.1)  m: 40.4 (13.0) | *W* = 142, *p* = .05* | f: 31.6 (11.0)  m: 34.1 (10.3) | *W* = 49.5, *p* = .51 | f: 36.5 (12.9)  m: 34.3 (11.0) | *W* = 674, *p* = .47 |
| BMI | f: 22.4 (4.5)  m: 25.2 (3.0) | *W* = 120, *p* = .01* | f: 26.0 (6.1)  m: 24.5 (2.9) | *W* = 64, *p* = .82 | f: 25.7 (5.5)  m: 25.1 (4.3) | *W* = 651, *p* = .66 |
| Education | f: 16.2 (3.0)  m: 16.4 (3.8) | *W* = 221, *p* = 1 | f: 12.4 (1.8)  m: 13.5 (2.1) | *W* = 34.5, *p* = .09 | f: 13.2 (2.2)  m: 12.8 (2.5) | *W* = 676, *p* = .45 |
| Medication (OLZ eq.) | - | - | f: 15.9 (10.7)  m: 12.5 (10.3) | *W* = 76.5, *p* =.29 | f: 15.4 (10.4)  m: 19.1 (10.9) | *W* = 490, *p* = .15 |
| Duration of illness | - | - | f: 5.4 (5.7)  m: 7.9 (9.1) | *W* = 56, *p* = .82 | f: 9.9 (9.9)  m: 9.7 (9.1) | *W* = 602, *p* = .90 |
| Number of episodes |  |  | f: 3.3 (1.7)  m: 3.4 (3.4) | *W* = 64, *p* = .54 | f: 5.2 (4.1)  m: 4.8 (5.3) | *W* = 674, *p* = .47 |
| B) Clinical scales | | | | | | |
| PANSS total | - | - | f: 66.3 (13.6)  m: 63.5 (16.5) | *W* = 70, *p* = .53 | f: 81.1 (14.8)  m: 78.7 (17.8) | *W* = 704, *p* = .29 |
| PANSS neg. | - | - | f: 15.3 (3.9)  m: 15.0 (4.5) | *W* = 556, *p* = .51 | f: 23.2 (6.3)  m: 24.2 (6.1) | *W* = 64.5, *p* = .79 |
| PANSS pos. | - | - | f: 16.6 (5.1)  m: 15.8 (4.3) | *W* = 64.5, *p* = .79 | f: 16.4 (4.0)  m: 15.6 (6.0) | *W* = 713, *p* = .24 |
| SRRS | - | - | f: 8.9 (3.2)  m: 7.7 (2.3) | *W* = 74.5, *p* = .35 | f: 23.2 (5.6)  m: 23.6 (5.7) | *W* = 597, *p* = .86 |
| mSRRS | - | - | f: 3.5 (2.0)  m: 2.1 (0.9) | *W* = 87.5, *p* = .06 | f: 10.3 (3.2)  m: 10.6 (2.8) | *W* = 543, *p* = .41 |
| BFCRS | - | - | f: 1.2 (0.9)  m: 1.4 (2.4) | *W* = 70.5, *p* = .49 | f: 5.1 (3.9)  m: 5.5 (4.2) | *W* = 584, *p* = .74 |
| UPDRS | - | - | f: 9.1 (6.4)  m: 7.8 (5.3) | *W* = 66, *p* = .72 | f: 18.4 (10.5)  m: 22.1 (11.5) | *W* = 495, *p* = .17 |
| BNSS | - | - | f: 28.0 (12.0)  m: 23.9 (8.1) | *W* = 75.5, *p* = .32 | f: 39.8 (13.8)  m: 43.6 (14.5) | *W* = 507, *p* = .22 |
| BNSS Expressivity | - | - | f: 2.0 (1.0)  m: 1.4 (1.0) | *W* = 83.5, *p* = .13 | f: 3.4 (1.4)  m: 3.6 (1.3) | *W* = 578, *p* = .69 |
| BNSS Avolition | - | - | f: 2.8 (1.5)  m: 2.5 (1.0) | *W* = 71.5, *p* = .46 | f: 3.2 (1.6)  m: 3.3 (1.5) | *W* = 603, *p* = .91 |
| C) Gait parameters | | | | | | |
| Velocity (cm/s) | f: 130 (16.6)  m:124 (18.4) | *W* = 258, *p* = .36 | f: 110 (12.6)  m: 123 (17.6) | *W* = 36, *p* = .12 | f: 104 (17.8)  m: 105 (24.8) | *W* = 600, *p* = .88 |
| Cadence (footfalls/min) | f: 116 (9.8)  m: 109 (8.1) | *W* = 314, *p* = .018* | f: 108 (5.6)  m: 109 (10.5) | *W* = 52, *p* = .63 | f: 105 (10.5)  m: 101 (12.7) | *W* = 686, *p* = .39 |
| Stride Length (cm) | f: 134 (11.6)  m: 136 (15.1) | *W* = 202, *p* = .65 | f: 123 (9.6)  m: 135 (10.3) | *W* = 22, *p* = .011* | f: 118 (12.4)  m: 124 (18.0) | *W* = 484, *p* = .13 |
| FAP | f: 89.6 (13.2)  m: 96.6 (3.2) | *W* = 149, *p* = .07 | f: 98.5 (1.1)  m: 96.7 (3.4) | *W* = 67, *p* = .67 | f: 96.0 (5.0)  m: 88.1 (17.9) | *W* = 723, *p* = .20 |
| Variance in Stride Time | f: 1.80 (0.6)  m: 2.02 (1.6) | *W* = 251, *p* = .45 | f: 2.19 (1.1)  m: 1.50 (0.31) | *W* = 93, *p* = .030* | f: 2.26 (1.1)  m: 2.48 (1.5) | *W* = 580, *p* = .71 |
| Variance in Stride Length | f: 2.05 (0.70)  m: 1.68 (0.45) | *W* = 284, *p* = .11 | f: 2.09 (0.62)  m: 1.89 (0.79) | *W* = 73, *p* = .42 | f: 2.28 (1.2)  m: 2.6 (1.6) | *W* = 577, *p* = .68 |

*Note*: For each measure the mean is indicated with the standard deviation (SD) in brackets separately for female and male participants. For comparison between sexes a Mann-Whitney-U-Test is performed (W). P-values are uncorrected.

N: number of participants; f: female; m: male; BMI: Body Mass Index; OLZ eq.: Olanzapine-equivalent (mg/day); PANSS: Positive And Negative Syndrome Scale; neg.: negative; pos.: positive; SRRS: Salpêtrière Retardation Rating Scale; mSRRS: motoric part of the Salpêtrière Retardation Rating Scale; BFCRS: Bush-Francis Catatonia Rating Scale; UPDRS: Unified Parkinson Disease Rating Scale Part III; BNSS: Brief Negative Symptom Scale. FAP: functional ambulation performance score.

** p* ≤ .05.

Table S2: Test for equal distribution of BMI categories between groups

|  |  | Group | | | Comparison |
| --- | --- | --- | --- | --- | --- |
|  |  | HC | NPS | PS | p-value |
| BMI | Underweight (< 18.5) | 4 | 1 | 2 | .51 |
|  | Normal-weight (18.5 – < 25) | 21 | 11 | 35 |  |
|  | Overweight (25 – < 30) | 15 | 8 | 22 |  |
|  | Obesity (>= 30) | 2 | 2 | 11 |  |

*Note*: For each cell, the number of participants is indicated. For comparison between groups a Fisher’s exact test for count data is performed (alternative = two-sided).

BMI: Body Mass Index; HC: healthy controls; NPS: non-psychomotor slow; PS: psychomotor slow.

** p* ≤ .05.

Table S3: Effects of Group, Condition, and Sex on Gait Performance for each Gait Parameter

|  | Interaction effects | | Main Effects | | Covariates | |
| --- | --- | --- | --- | --- | --- | --- |
| Velocity | Group * Condition * Sex | *F*(6,384) = 0.7, *p* = .63 | Groups | *F*(2,126) = 35.6, *p* < .001* | BMI | *F*(1,126) = 2.2, *p* = .14 |
|  | Condition * Sex | *F*(3,384) = 0.7, *p* = .55 | Condition | *F*(3,384) = 1138, *p* < .001* | Age | *F*(1,126) = 2.2, *p* = .14 |
|  | Groups * Sex | *F*(2,126) = 0.4, *p* = .67 | Sex | *F*(1,126) = 0.04, *p* = .84 |  |  |
|  | Groups * Condition | *F*(6,384) = 8.0, *p* < .001* |  |  |  |  |
| Cadence | Group * Condition * Sex | *F*(6,384) = 0.2, *p* = .97 | Groups | *F*(2,126) = 22.7, *p* < .001* | Age | *F*(1,126) = 2.8, *p* = .10 |
|  | Condition * Sex | *F*(3,384) = 4.7, *p* = .003* | Condition | *F*(3,384) = 619, *p* < .001* | BMI | *F*(1,126) = 0.2, *p* = .69 |
|  | Groups * Sex | *F*(2,126) = 0.3, *p* = .71 | Sex | *F*(1,126) = 9.2, *p* = .003* |  |  |
|  | Groups * Condition | *F*(6,384) = 4.8, *p* < .001* |  |  |  |  |
| Stride Length | Group * Condition * Sex | *F*(6,384) = 0.7, *p* = .66 | Groups | *F*(2,126) = 17.3, *p* < .001* | Age | *F*(1,126) = 12.0, *p* < .001* |
|  | Condition * Sex | *F*(3,384) = 5.1, *p* = .002* | Condition | *F*(3,384) = 662, *p* < .001* | BMI | *F*(1,126) = 6.2, *p* = .014* |
|  | Groups * Sex | *F*(2,126) = 1.0, *p* = .37 | Sex | *F*(1,126) = 8.8, *p* = .004* |  |  |
|  | Groups * Condition | *F*(6,384) = 0.3, *p* = .96 |  |  |  |  |
| FAP | Group * Condition * Sex | *F*(4,256) = 0.5, *p* = .77 | Groups | *F*(2,126) = 6.0, *p* = .003* | Age | *F*(1,126) = 1.7, *p* = .19 |
|  | Condition * Sex | *F*(2,256) = 0.2, *p* = .83 | Condition | *F*(2,256) = 59.4, *p* < .001* | BMI | *F*(1,126) = 0.5, *p* = .47 |
|  | Groups * Sex | *F*(2,126) = 5.9, *p* = .004* | Sex | *F*(1,126) = 1.8, *p* = .18 |  |  |
|  | Groups * Condition | *F*(4,256) = 7.0, *p* < .001* |  |  |  |  |
| Variance in Stride Time | Group * Condition* Sex | *F*(6,384) = 0.6, *p* = .73 | Groups | *F*(2,126) = 5.2, *p* = .007* | Age | *F*(1,126) = 3.8, *p* = .05 |
|  | Condition * Sex | *F*(3,384) = 0.2, *p* = .87 | Condition | *F*(3,384) = 84.4, *p* < .001* | BMI | *F*(1,126) = 0.02, *p* = .88 |
|  | Groups * Sex | *F*(2,126) = 0.9, *p* = .41 | Sex | *F*(1,126) = 1.7, *p* = .19 |  |  |
|  | Groups * Condition | *F*(6,384) = 3.8, *p* = .001* |  |  |  |  |
| Variance in Stride Length | Group * Condition * Sex | *F*(6,384) = 0.5, *p* = .81 | Groups | *F*(2,126) = 7.2, *p* = .001* | Age | *F*(1,126) = 2.5, *p* = .11 |
|  | Condition * Sex | *F*(3,384) = 0.2, *p* = .90 | Condition | *F*(3,384) = 287, *p* < .001* | BMI | *F*(1,126) = 2.3, *p* = .13 |
|  | Groups * Sex | *F*(2,126) = 2.0, *p* = .14 | Sex | *F*(1,126) = 0.1, *p* = .79 |  |  |
|  | Groups * Condition | *F*(6,384) = 2.8, *p* = .010* |  |  |  |  |

*Note*: For each comparison (btw. 3 groups, 4 conditions, 2 genders) an ANCOVA is performed, controlled for age and BMI. For FAP there are only 3 conditions included. P-values are uncorrected for multiple comparison.

f: female; m: male; BMI: Body Mass Index; FAP: functional ambulation performance score.

** p* ≤ .05.

Table S4: Spearman Correlations between Gait Parameters and Clinical Scales across all Schizophrenia Patients (N = 92) controlled for Age, BMI, and Medication

|  | Condition | mSRRS | UPDRS | BFCRS |
| --- | --- | --- | --- | --- |
| Velocity | Self_Speed | rho = -.26, *p* = .015, *p_corr_* = .033* | rho = -.29, *p* = .006, *p_corr_* = .015* | rho = -.21, *p* = .053, *p_corr_* = .09 |
|  | Max_Speed | rho = -.43, *p* < .001, *p_corr_* < .001* | rho = -.51, *p* < .001, *p_corr_* < .001* | rho = -.35, *p* < .001, *p_corr_* = .003* |
| Cadence | Self_Speed: | rho = -.19, *p* = .07, *p_corr_* = .09 | rho = -.18, *p* = .10, *p_corr_* = .12 | rho = -.10, *p* = .35, *p_corr_* = .37 |
|  | Max_Speed | rho = -.33, *p* = .002, *p_corr_* = .005* | rho = -.38, *p* < .001, *p_corr_* = .002* | rho = -.33, *p* = .002, *p_corr_* = .005* |
| Stride Length | Self_Speed | rho = -.27, *p* = .01, *p_corr_* = .023* | rho = -.36, *p* < .001, *p_corr_* = .003* | rho = -.25, *p* = .020, *p_corr_* = .038* |
|  | Max_Speed | rho = -.19, *p* = .08, *p_corr_* = .10 | rho = -.21, *p* = .04, *p_corr_* = .07 | rho = -.12, *p* = .27, *p_corr_* = .30 |
| FAP | Self_Speed: | rho = -.20, *p* = .06, *p_corr_* = .09 | rho = -.13, *p* = .24, *p_corr_* = .28 | rho = -.06, *p* = .59, *p_corr_* = .59 |
|  | Max_Speed | - | - | - |

*Note*: Spearman correlations are calculated across all patients (70 PS and 22 NPS) and controlled for age, BMI, and medication (rho). All p-values are FDR corrected for multiple comparison (*p_corr_*).

mSRRS: motoric part of the Salpêtrière Retardation Rating Scale; UPDRS: Unified Parkinson Disease Rating Scale Part III; BFCRS: Bush-Francis Catatonia Rating Scale; Self_Speed: self-selected speed; Max_Speed: maximum speed; FAP: functional ambulation performance score.

*** *p_corr_* ≤ .05

Table S5: Spearman Correlations between Gait Parameters and BNSS plus Subdomains across all Schizophrenia Patients (N = 92)

|  | BNSS total | | Subdomain  Avolition | | Subdomain  Expressivity | |
| --- | --- | --- | --- | --- | --- | --- |
| Condition | Self_Speed | Max_Speed | Self_Speed | Max_Speed | Self_Speed | Max_Speed |
| Velocity | rho = -.27,  *p* = .010* | rho = -.32,  *p* = .002* | rho = -.26,  *p* = .013* | rho = -.22,  *p* = .036* | rho = -.20,  *p* = .07 | rho = -.32,  *p* = .002* |
| Cadence | rho = -.28,  *p* = .008* | rho = -.30,  *p* = .004* | rho = -.21,  *p* = .048* | rho = -.16,  *p* = .13 | rho = -.17,  *p* = .10 | rho = -.35,  *p* < .001* |
| Stride Length | rho = -.24,  *p* = .023* | rho = -.09,  *p* = .42 | rho = -.28,  *p* = .008* | rho = -.06,  *p* = .56 | rho = -.20,  *p* = .06 | rho = -.05,  *p* = .62 |
| FAP | rho = .03,  *p* = .80 | - | rho = .10,  *p* = .35 | - | rho = -.03,  *p* = .77 | - |

*Note*: Spearman correlations are calculated across all patients (70 PS and 22 NPS) and controlled for age, BMI, and medication (rho). As this analysis is exploratory, p-values are uncorrected for multiple comparison. N = number of participants; BNSS: Brief Negative Symptom Scale; Self_Speed: self-selected speed; Max_Speed: maximum speed; FAP: functional ambulation performance score.

** p* ≤ .05.

Table S6: Correlation of number of episodes and duration of illness with gait parameters during self-selected speed

|  | Velocity | Cadence | Stride Length |
| --- | --- | --- | --- |
| Number of episodes | rho = -.12, *p* = .25 | rho = -.07, *p* = .52 | rho = -.17, *p* = .10 |
| Duration of illness | rho = -.12, *p* = .25 | rho = -.07, *p* = .51 | rho = -.17, *p* = .10 |

*Note*: Spearman correlations are calculated across all patients (70 PS and 22 NPS) (rho). As this analysis is exploratory, p-values are uncorrected for multiple comparison.

NPS: non-psychomotor slow; PS: psychomotor slow.

** p* ≤ .05.

Table S7: Effects of duration of illness and number of episodes on the ANCOVA patients

|  | **ANCOVA patients**  (controlled for age, BMI, Medication) | **Additional ANCOVA patients**  (controlled for age, BMI, Medication, duration of illness, number of episodes) |
| --- | --- | --- |
| Velocity (cm/s) | *Interaction: Condition*Group*  *F*(3,270) = 5.9, *p* < .001  *Main effects:*  Groups: *F*(1,87) = 14.0, *p <* .001*  Condition*: F*(3, 270) = 717, *p* < .001*  *Covariates:*  Age: *F*(1,87) = 3.8, *p* = .055  BMI: *F*(1,87) = 1.2, *p* = .28  Medication: *F*(1,87) = 1.4, *p* = .24 | *Interaction: Condition*Group*  *F*(3,267) = 6.28, *p* < .001*  *Main effects:*  Groups: *F*(1,84) = 17.5, *p <* .001*  Condition*: F*(3,267) = 713, *p* < .001*  *Covariates:*  Age: *F*(1,84) = 2.9, *p* = .09  BMI: *F*(1,84) = 0.7, *p* = .40  Medication: *F*(1,84) = 1.4, *p* = .24  Duration of Illness:  *F*(1,84) = 2.3, *p* = .14  Number of Episodes:  *F*(1,84) = 0.2, *p* = .65 |
| Cadence (footfalls/min) | *Interaction: Condition*Group*  *F*(6,393) = 4.7, *p* < .001  *Main effects:*  Group: *F*(2,129) = 21.4, *p* < .001*  Condition*: F*(3,393) = 610, *p* < .001*  *Covariates:*  Age: *F*(1,129) = 2.0, *p* = .16  BMI: *F*(1,129) = 0.1, *p* = .72  Medication: *F*(1,87) = 4.3, *p* = .042* | *Interaction: Condition*Group*  *F*(3,267) = 4.5, *p* = .004*  *Main effects:*  Groups: *F*(1,84) = 13.2, *p <* .001*  Condition*: F*(3,267) = 414, *p* < .001*  *Covariates:*  Age: *F*(1,84) = 0.5, *p* = .50  BMI: *F*(1,84) = 1.3, *p* = .26  Medication: *F*(1,84) = 4.4, *p* = .04*  Duration of Illness:  *F*(1,84) = 3.8, *p* = .054  Number of Episodes:  *F*(1,84) = 0.3, *p* = .58 |
| Stride Length (cm) | *Interaction: Condition*Group*  *F*(6,393) = 0.2, *p* = .96  *Main effects:*  Group: *F*(2,129) = 16.2, *p* < .001*  Condition*: F*(3,393) = 645, *p* < .001*  *Covariates:*  Age: *F*(1,129) = 9.9, *p* = .002*  BMI: *F*(1,129) = 5.7, *p* = .019*  Medication: *F*(1,87) = 0.002, *p* = .96 | *Interaction: Condition*Group*  *F*(3,267) = 0.14, *p* = .94  *Main effects:*  Groups: *F*(1,84) = 6.3, *p =* .014*  Condition*: F*(3,267) = 439, *p* < .001*  *Covariates:*  Age: *F*(1,84) = 7.6, *p* = .007*  BMI: *F*(1,84) = 5.1, *p* = .027*  Medication: *F*(1,84) = 0.005, *p* = .94  Duration of Illness:  *F*(1,84) = 0.3, *p* = .61  Number of Episodes:  *F*(1,84) = 1.3, *p* = .26 |

*Note*: ANCOVA patients: analysing the effect of only patient groups (NPS vs. PS) and 4 conditions on gait controlling for age, BMI, and medication. Additional ANCOVA patients: adding the covariates number of episodes and duration of illness in the previous ANCOVA.

BMI: Body Mass Index; sd: standard deviation; PS: psychomotor slow; NPS: non-psychomotor slow; HC: healthy controls.

** p* ≤ .05

Table S8: Effect of Group on Gait Performance for each Gait Parameter during self-selected Speed

|  | ANCOVA | Mean (SD) | Contrasts |
| --- | --- | --- | --- |
| Velocity | Group: *F*(2) = 17.7, *p* < .001*  Age: *F*(1) = 1.4, *p* = .23  BMI: *F*(1) = 0.4, *p* = .52 | HC: 127 (17.6)  NPS: 116 (16.0)  PS: 104 (21.5) | PS vs. HC: *p* < .001*  NPS vs. HC: *p* = .11  PS vs. NPS: *p* = .044* |
| Cadence | Group: *F*(2) = 11.3, *p* < .001*  Age: *F*(1) = 0.2, *p* = .69  BMI: *F*(1) = 1.2, *p* = .29 | HC: 113 (9.65)  NPS: 109 (8.04)  PS: 103 (11.8) | PS vs. HC: *p* < .001*  NPS vs. HC: *p* = .31  PS vs. NPS: *p* = .06 |
| Stride Length | Group: *F*(2) = 14.0, *p* < .001*  Age: *F*(1) = 5.1, *p* =.026*  BMI: *F*(1) = 3.9, *p* = .049* | HC: 135 (13.3)  NPS: 128 (11.5)  PS: 121 (15.6) | PS vs. HC: *p* < .001*  NPS vs. HC: *p* = .16  PS vs. NPS: *p* = .12 |

*Note*: For each comparison (btw. 3 groups) an ANCOVA is performed, controlled for age and BMI. P-values are uncorrected for multiple comparison. For each measure the mean is indicated with the standard deviation (SD) in brackets. Contrasts are automatically Tukey corrected for multiple comparison within each model.

BMI: Body Mass Index; PS: psychomotor slow; NPS: non-psychomotor slow; HC: healthy controls.

** p* ≤ .05.

Table S9: Effect of Condition on Gait Performance for each Gait parameter separately for each Group

| A) ANCOVA | | | | | | | | | |
| --- | --- | --- | --- | --- | --- | --- | --- | --- | --- |
|  |  | Velocity | | Cadence | | | Stride Length | | |
| Group | Condition | Mean (SD) | ANCOVA | Mean (SD) | | ANCOVA | Mean (SD) | | ANCOVA |
| HC  (*N* = 42) | Self_Speed | 127 (17.6) | Condition  *F*(3) = 308, *p* < .001*  Covariates  Age: *F*(1) = 0.1, *p* = .71  BMI: *F*(1) = 5.0, *p* = .027* | 113 (9.65) | | Condition  *F*(3) = 155, *p* < .001*  Covariates  Age: *F*(1) = 3.6, *p* = .06  BMI: *F*(1) = 0.105, *p* = .75 | 135 (13.3) | | Condition  *F*(3) = 109, *p* < .001*  Covariates  Age: *F*(1) = 1.6, *p* = .21  BMI: *F*(1) = 4.9, *p* = .029* |
|  | Max_Speed | 237 (26.7) |  | 168 (22.2) | |  | 171 (19.5) | |  |
|  | Head_Rec | 114 (25.5) |  | 109 (14.0) | |  | 125 (15.4) | |  |
|  | Eyes_Closed | 97.6 (23.8) |  | 107 (13.1) | |  | 109 (17.6) | |  |
| NPS  (*N* = 22) | Self_Speed | 116 (16.0) | Condition  *F*(3) = 144, *p* < .001*  Covariates  Age: *F*(1) = 1.3, *p* = .25  BMI: *F*(1) = 5.1, *p* = .027* | 109 (8.04) | | Condition  *F*(3) = 77.7, *p* < .0001*  Covariates  Age: *F*(1) = 0.002, *p* = .96  BMI: *F*(1) = 1.9, *p* = .17 | 128 (11.5) | | Condition  *F*(3) = 51.2, *p* < .001*  Covariates  Age: *F*(1) = 2.3, *p* = .13  BMI: *F*(1) = 5.4, *p* = .023* |
|  | Max_Speed | 221 (31.2) |  | 163 (22.0) | |  | 165 (24.2) | |  |
|  | Head_Rec | 104 (28.7) |  | 106 (16.5) | |  | 117 (18.8) | |  |
|  | Eyes_Closed | 84.2 (18.7) |  | 101 (11.6) | |  | 99.7 (17.5) | |  |
| PS  (*N* = 70) | Self_Speed | 104 (21.5) | Condition  *F*(3) = 282, *p* < .001*  Covariates  Age: *F*(1) = 7.2, *p* = .007*  BMI: *F*(1) = 0.4, *p* = .51 | 103 (11.8) | | Condition  *F*(3) = 164, *p* < .001*  Covariates  Age: *F*(1) = 1.7, *p* = .20  BMI: *F*(1) = 2.2, *p* = .14 | 121 (15.6) | | Condition  *F*(3) = 165, *p* < .001*  Covariates  Age: *F*(1) = 26.3, *p* < .001*  BMI: *F*(1) = 6.4, *p* = .012* |
|  | Max_Speed | 187 (30.2) |  | 145 (19.4) | |  | 156 (20.0) | |  |
|  | Head_Rec | 94.3 (26.9) |  | 101 (15.7) | |  | 111 (18.9) | |  |
|  | Eyes_Closed | 71.8 (21.6) |  | 92.8 (13.6) | |  | 91.8 (18.6) | |  |
| B) Contrasts | | | | | | | | | |
| Group | Comparison | | Velocity | | Cadence | | | Stride Length | |
| HC  (*N* = 42) | Self_Speed - Max_Speed | | Diff = -110.8, *p* < .001* | | Diff = -55.72, *p* < .001* | | | Diff = -36.1, *p* < .001* | |
|  | Self_Speed - Head_Rec | | Diff = 12.8, *p* = .06 | | Diff = 3.91, *p* = .65 | | | Diff = 10.3, *p* = .024* | |
|  | Self_Speed - Eyes_Closed | | Diff = 29.1, *p* < .001* | | Diff = 5.41, *p* = .38 | | | Diff = 26.4, *p* < .001* | |
|  | Max_Speed - Head_Rec | | Diff = 123.5, *p* < .001* | | Diff = 59.63, *p* < .001* | | | Diff = 46.3, *p* < .001* | |
|  | Max_Speed - Eyes_Closed | | Diff = 139.8, *p* < .001* | | Diff = 61.12, *p* < .001* | | | Diff = 62.5, *p* < .001* | |
|  | Head_Rec - Eyes_Closed | | Diff = 16.3, *p* = .005* | | Diff = 1.49, *p* = .99 | | | Diff = 16.1, *p* < .001* | |
| NPS  (*N* = 22) | Self_Speed - Max_Speed | | Diff = -104.7, *p* < .001* | | Diff = -54.18, *p* < .001* | | | Diff = -36.5, *p* < .001* | |
|  | Self_Speed - Head_Rec | | Diff = 11.9, *p* = .36 | | Diff = 3.13, *p* = 91 | | | Diff = 11.2, *p* = .17 | |
|  | Self_Speed - Eyes_Closed | | Diff = 32.0, *p* < .001* | | Diff = 7.5, *p* = .38 | | | Diff = 28.4, *p* < .001* | |
|  | Max_Speed - Head_Rec | | Diff = 116.6, *p* < .001* | | Diff = 57.3, *p* < .001* | | | Diff = 47.7, *p* < .001* | |
|  | Max_Speed - Eyes_Closed | | Diff = 136.6, *p* < .001* | | Diff = 61.68, *p* < .001* | | | Diff = 64.9, *p* < .001* | |
|  | Head_Rec - Eyes_Closed | | Diff = 20.1, *p* = .033* | | Diff = 4.38, *p* = .78 | | | Diff = 17.2, *p* = .011* | |
| PS  (*N* = 70) | Self_Speed - Max_Speed | | Diff = -82.8, *p* < .001* | | Diff = -42.18, *p* < .001* | | | Diff = -34.85, *p* < .001* | |
|  | Self_Speed - Head_Rec | | Diff = 9.9, *p* = .09 | | Diff = 2.31, *p* = .81 | | | Diff = 9.69, *p* = .006* | |
|  | Self_Speed - Eyes_Closed | | Diff = 32.4, *p* < .001* | | Diff = 9.94, *p* < .001* | | | Diff = 29.06, *p* < .001* | |
|  | Max_Speed - Head_Rec | | Diff = 92.7, *p* < .001* | | Diff = 44.49, *p* < .001* | | | Diff = 44.55, *p* < .001* | |
|  | Max_Speed - Eyes_Closed | | Diff = 115.1, *p* < .001* | | Diff = 52.12, *p* < .001* | | | Diff = 63.92, *p* < .001* | |
|  | Head_Rec - Eyes_Closed | | Diff = 22.5, *p* < .001* | | Diff = 7.63, *p* = .019* | | | Diff = 19.37, *p* < .001* | |

*Note*: A) For each comparison (btw. 4 condition) an ANCOVA is performed, controlled for age and BMI. P-values are uncorrected for multiple comparison. For each measure the mean is indicated with the standard deviation (SD) in brackets.

B) Contrasts are automatically Tukey corrected for multiple comparison within each model.

BMI: Body Mass Index; HC: healthy controls; NPS: non-psychomotor slow; PS: psychomotor slow; N: number of participants; Self_Speed: self-selected speed; Max_Speed: maximum speed; Head_Rec: head reclination; Eyes_Closed: eyes closed; Diff: difference.
